# Embryogenesis of bladder exstrophy: A new hypothesis

**DOI:** 10.4103/0971-9261.43017

**Published:** 2008

**Authors:** Bharati Kulkarni, Navin Chaudhari

**Affiliations:** Department of Paediatric Surgery, Lokmanya Tilak Municipal General Hospital, Sion, Mumbai - 400 022, India

**Keywords:** Bladder exstrophy, embryology of cloaca, genital tubercle

## Abstract

**Aims and Objective:**

To postulate a hypothesis to explain the embryogenesis of exstrophy bladder based on our clinical observations.

**Materials and Methods:**

In 27 cases of exstrophy, we measured the distance between the lowermost inguinal skin crease to the root of the penis (clitoris) (B) and the distance between the penis (clitoris) and the scrotum (labia majora) (C). These were compared with age, height and XP distance (distance between xiphisternum and symphysis pubis) matched control group of normal children. The distance between the lowermost inguinal skin crease and the penis (clitoris) (A) was measured in control group.

**Results:**

The observation was A = B + C. This implies that in exstrophy bladder, the position of the penis (clitoris) has moved cephalad from the lower border of A to the junction of B and C.

**Conclusion:**

Based on the observations, we postulate that abnormal origin of genital tubercle may be the cause of exstrophy bladder. The abnormal origin of primordia of the genital tubercle in more cephalad direction than normal causes wedge effect, which will interfere with the medial migration of the mesoderm as well as the midline approximation of mesodermal structures in the lower abdominal wall, thereby resulting in the exstrophy of bladder.

## INTRODUCTION

The anomaly of exstrophy bladder not only involves the urinary tract and genital system but also the musculoskeletal system of lower abdomen and pelvis. Various theories have been proposed to explain the embryogenesis of this crippling condition. We postulate a hypothesis on the embryogenesis of exstrophy bladder based on our clinical findings, Normally, the darker skin of the penis and the scrotum are contiguous. In exstrophy, the penile and the scrotal skin appear to be interposed by a band of skin of some breadth with little or no pigmentation. Reconstructing the story in retrospective manner from this telltale evidence, we propose that the exstrophy of the bladder is the result of the cephalad origin of genital tubercle. This in turn will have a wedge effect and produce interference with the medial migration of the mesoderm and prevent cloacal migration in both the dorsal and caudal direction.

Many congenital anomalies correspond to the normal embryonic stage, that is, the eventual deformity is essentially present at some stage in the normal embryo.[1–3] This signifies developmental arrest as the cause of the anomaly.[4,5] On the other hand, exstrophy bladder is never present as a normal developmental stage nor has been recognized in abnormal embryos. Many congenital anomalies, particularly those resulting from developmental arrest, have been experimentally produced but not exstrophy of bladder. This peculiarity suggests that exstrophy is not caused by mere developmental arrest. Here, we have made an effort to explain the embryological basis for genesis of exstrophy bladder on the basis of our clinical observations.

## MATERIALS AND METHODS

On our record, we have 68 cases of exstrophy bladder, 51 male and 17 female children. The age group ranged from 1 day to 23 years. In all these cases of exstrophy bladder, the presence of a normally pigmented skin bridge that dissociated penile shaft from scrotum was noted. Earlier, sufficient attention has not been paid to this skin bridge. A similar skin bridge was present in female patients between the clitoris and the labia majora.

**Figure 1:**
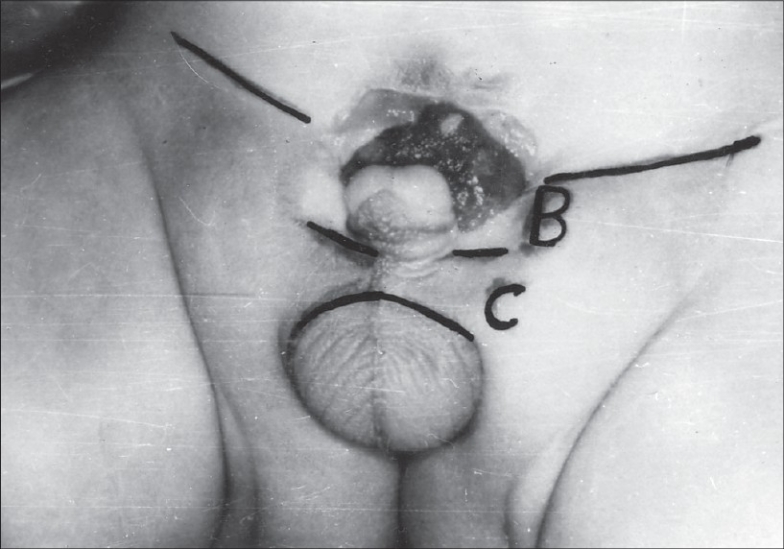
Distance between the lowest inguinal skin crease and the root of penis. Distance between the root of penis and the upper border of scrotum

**Figure 2:**
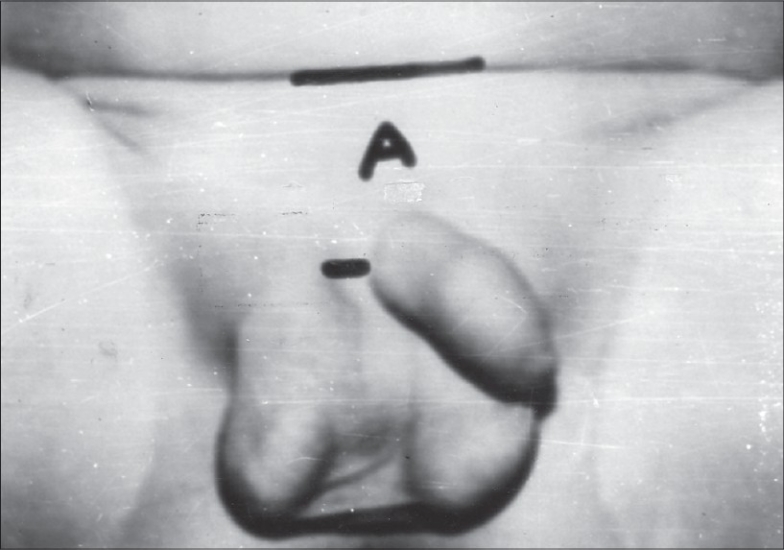
Distance between the lowest inguinal skin crease and penis

In 27 cases (21 male and 6 female children) we measured the distance between the lowest inguinal skin crease and the root of penis (clitoris in female) (B) and the root of penis (clitoris) and the upper border of scrotum (labia majora) (C) [Figure 1]. Then, this was compared with the distance between the lowest inguinal skin crease and the penis (clitoris) (A) in the control group [Figure 2]. The control group was selected by matching age, height and XP (distance between the xiphisternum and the pubic symphisis). The height of patients with exstrophy bladder was below the tenth percentile according to I.C.M.R. chart. Hence, the selection of control group matching these three factors was a tedious job.

## RESULTS

**Table:** Demographical data

Parameters	Range for control and study
Age	1 day-23 years
Height in cm	43-165
XP Distance in cm	10.8-35

All the study cases were matched with the control group for age, height and XP distance.

Comparison of the (B + C) distance in exstrophy cases with distance A in control group

**Table:** 

Group	Average distance (X – SD) cm
Control (A)	3.878 + 0.839
Cases (B + C)	3.541 + 0.730

The data given above reveals that the average distance of the lowest inguinal skin crease to the root of the penis in the control group was 3.878 cm, which is almost same as the distance (B + C) of the disease cases, i.e., 3.541 cm. The difference between these was not significant statistically. The analysis was done by student's *t* test.

The distance between the inguinal skin crease and the penis (clitoris) (A) in the control group corresponds to the sum of the distance between the inguinal skin crease and the penis (clitoris) (B) and the distance between the penis (clitoris) and the scrotum (labia majora) (C). In children with exstrophy of bladder, A = B + C. Two of the boys and one girl with exstrophy were post-pubertal in our series. In these cases, the distribution of pubic hair could be observed. It was observed that the growth of pubic hair discontinues at the level of penis (clitoris) and grows downwards and medially under the penis (clitoris) instead of growing superomedially.

## DISCUSSION

In the normal embryo between the second and third week after fertilization, just caudal to the primitive streak, the opposition of the ectoderm and endoderm takes place in the midline without the in-growth of the mesoderm forming a bilaminar epithelial layer called the cloacal membrane. By the fourth week, due to rapid longitudinal growth of the embryo, this caudal area elongates and curves so that the cloacal membrane occupies a position ventrally, caudal to body stalk, forming the ventral wall of the urogenital sinus. Early in the fourth week, paired primordia of the genital tubercle develop in both the sexes at the cranial end of the cloacal membrane. The genital tubercle soon enlarges to form a phallus, which is as large in female as in male embryo at this indifferent stage. The growth of genital tubercle is accompanied by the migration of mesoderm towards midline, thereby lengthening the area between the body stalk and the cloacal membrane. At the same time the urorectal fold grows medially and caudally towards the primitive perineum, dividing the urogenital sinus from the rectum.

The embryogenesis of exstrophy-epispadias complex is a subject that has been a source of conjecture and controversy. It is also known that the human embryo does not pass through a stage of development that corresponds to exstrophy bladder. Hence, it is impossible from embryologic point of view to explain the bladder exstrophy as a result of simple developmental arrest.[6,7] Numerous other theories have been proposed to explain the etiopathogenesis of exstrophy bladder. "Bersting's Theory" states that the anterior rupture of the embryonic bladder is caused by the abnormal retention of fluid.[8] However, this does not explain the commonly associated anomalies of genital and musculoskeletal systems. Pattern and Barry theorized that the caudal displacement of the genital tubercle is attributable for exstrophy bladder.[9] We refute this theory supporting the explanation of Marshall and Muecke that if the genital tubercle is displaced caudally, one might expect, with more severe degree of exstrophy to find the corpora located on the perineum or dissociated from their bony attachment, which rarely occurs.[10]

Marshall and Muecke proposed that the abnormally large cloacal membrane would act as a wedge to the developing structure of the lower abdominal wall.[6] We agree with Marshall and Muecke with respect to their conclusion that the wedge effect is preventing the medial migration; however, the wedge is not due to cloacal membrane. We feel that cloacal membrane is a normal embryologic structure in that area, and even if it is abnormally large, it should not act as a wedge. A cloacal membrane normally is an unstable structure lacking mesoderm, and it has a strong tendency to disintegrate. Hence, it can not have wedge effect.

Thomalla *et al*.[11] have developed a model for the induction of cloacal exstrophy in the embryonic chick by use of the CO_2_ laser. The injury appears to cause a cellular defect in the primordial cloacal membrane. Even during the normal development, the cloacal membrane disintegrates at the seventh week. If the membrane is not supported by mesodermal rotation, the disintegration of this membrane will result in various components of the exstrophy-epispadias complex.

Based on the study of Wistar rats, Mildenberger *et al*.[12] proposed a hypothesis that the abnormal persistence of the caudal position of the insertion of the body stalk on the embryo causes exstrophy bladder. However, the embryogenesis of the rat in this area differs from that in humans, e.g., the human allantois apparently does not take part in the formation of the bladder, whereas in the rat it does. The second reason to disagree with the hypothesis put forward by Mildenberger *et al*.[12] that the growth of the genital tubercle is accompanied by the migration of the mesoderm towards the midline, thereby lengthening the area between the body stalk and the cloacal membrane. Thus, the persistence of low insertion of the body stalk is the effect of failure of the medial migration of the mesoderm and not the cause. Smith has proposed the theory of deformation as the mechanism for the abnormal development of embryogenesis. According to the theory, in the early stage of development the chorionic capsule and amniotic membrane that surround the embryo are physically stronger than the gel-like tissues of embryo itself. Any loss of amniotic fluid can subject the embryo to physical forces that may result in deformation of development. Such a deformation involving the caudal part of the early embryo could lead to any of the abnormal developmental mechanism, as suggested by Meuke, Mildenerger or Patten and Berry.

Beudoin *et al*.[13] have suggested the anatomical basis of a common embryological origin for epispadias, bladder and cloacal exstrophy. They suggest that the first anomaly could be the lack of rotation in the pelvic ring primordia. It is a fact that the pelvic ring primordia do not rotate; however, according to us, it is the result of the higher origin of the genital tubercle. The event of the rotation of pelvic ring primordia occurs later in a chronological order (seventh week) than during the origin of genital tubercle (fourth week).

The external genitalia pass through an undifferentiated or sexless state before the distinguishing sexual characteristics appear. Early in the fourth week, genital primordial develop in both the sexes at the cranial end of the cloacal membrane. These primordia are strong structures with mesenchyme. We propose that the abnormal origin of the primordia of the genital tubercle in a more cephalad position than the normal position will lead to wedge effect, and hence, it will interfere with the medial migration of the mesoderm as well as the midline approximation of the mesodermal structures in the lower abdominal wall and prevent the lengthening of the body stalk, which is observed in newborn babies whose umbilical cord is inserted at the apex of the exstrophied bladder. Our hypothesis is supported by the observation that in all the cases of exstrophy, there is a normally pigmented skin bridge interposed in between the penile shaft (clitoris in female) and the scrotum (labia majora in female) dissociating these two structures. It is proved by the fact that the distance A = B + C. The origin of genital tubercle is at the lower end of A in the control group. It has moved up in the cephalad direction at the junction of B and C in the study group. This is also supported by observing the pubic hair growth pattern in three of the post-pubertal patients with exstrophy. The hair is lacking in the symphyseal region and distributed in the sub-penile (sub-clitoral) area. This indicates that the penis (clitoris) is located superior to the skin of the pubic area. Thus, the abnormal position of the genital tubercle by even few microns in the cranial direction in a 4 mm embryo at 4 weeks of gestation will definitely have the wedge effect.

This is true for both the sexes as at an indifferent stage of the embryo (4-7 weeks), the genital tubercle that elongates to form phallus is as large in female as in male. The various components of the exstrophy-epispadias complex may be effectively explained by the incomplete rupture of the membrane at various anatomic levels, depending on the site of origin of genital tubercle.

Although this hypothesis of the wedge effect of genital tubercle causing exstrophy bladder seems to be interesting and logical, it will require further study on the embryos by moving the genital tubercle in the cephalad direction for strengthening it.

## CONCLUSIONS

We propose that abnormal origin of the primordia of genital tubercle in a cephalad position than the normal will result in the wedge effect, and hence, it will interfere with the medial migration of the mesoderm as well as the midline approximation of mesodermal structures in the lower abdominal wall and prevent the lengthening of the body stalk, which is observed in newborn babies whose umbilical cord is inserted at the apex of the exstrophied bladder. Our hypothesis is supported by the observation that in all cases of exstrophy, there is a normally pigmented skin bridge interposed in between the penile shaft (clitoris in female) and the scrotum (labia majora in female) dissociating these two structures.
